# Obstetric and perinatal outcomes in IVF versus ICSI-conceived pregnancies at a tertiary care center - a pilot study

**DOI:** 10.1186/1477-7827-11-84

**Published:** 2013-08-31

**Authors:** Kazem Nouri, Johannes Ott, Lucia Stoegbauer, Detlef Pietrowski, Sophie Frantal, Katharina Walch

**Affiliations:** 1 Department of Obstetrics and Gynecology, Medical University of Vienna, Vienna, Austria; 2 Center for Medical Statistics, Informatics and Intelligent Systems, Section for Medical Statistics of the Medical University of Vienna, Vienna, Austria

**Keywords:** IVF, ICSI, Pregnancy course, Obstetric outcome, Perinatal outcome

## Abstract

**Background:**

Although most pregnancies after IVF result in normal healthy outcomes, an increased risk for a number of obstetric and neonatal complications, compared to naturally conceived pregnancies, has been reported. While there are many studies that compare pregnancies after assisted reproductive techniques with spontaneously conceived pregnancies, fewer data are available that evaluate the differences between IVF and ICSI-conceived pregnancies. The aim of our present study was, therefore, to compare obstetric and perinatal outcomes in pregnancies conceived after in vitro fertilization (IVF) versus intracytoplasmatic sperm injection (ICSI).

**Methods:**

Three-hundred thirty four women who had become pregnant after an IVF or ICSI procedure resulted in a total of 530 children referred between 2003 und 2009 to the Department of Obstetrics and Gynecology of the Medical University of Vienna, a tertiary care center, and were included in this retrospective cohort study. We assessed maternal and fetal parameters in both groups (IVF and ICSI). The main study outcomes were preterm delivery, the need for neonatal intensive care, and congenital malformations. Moreover, we compared the course of pregnancy between both groups and the occurrence of complications that led to maternal hospitalization during pregnancy.

**Results:**

There were 80 children conceived via ICSI and 450 children conceived via IVF.

Mean gestational age was significantly lower in the ICSI group (p = 0.001). After ICSI, the birth weight (p = 0.008) and the mean APGAR values after 1 minute and after 10 minutes were lower compared to that of the IVF group (p = 0.016 and p = 0.047, respectively). Moreover, ICSI-conceived children had to be hospitalized more often at a neonatal intensive care unit (p = 0.004). There was no difference in pH of the umbilical artery or in major congenital malformations between the two groups. Pregnancy complications (i.e., premature rupture of membranes, cervical insufficiency, and premature uterine contractions) and the need for maternal hospitalization during pregnancy were found significantly more often after IVF (p = 0.0016 and p = 0.0095, respectively), compared to the ICSI group.

**Conclusions:**

When comparing IVF versus ICSI-conceived pregnancies at a tertiary care center, we found the course of pregnancy to be more complicated after IVF, whereas the primary fetal outcome seemed to be better in this group than after ICSI treatment.

## Background

In many countries, there is a tendency for more and more women to delay childbearing until an age when their fertility begins to decline. Therefore, in vitro fertilization (IVF) procedures have become accepted as an alternative to natural conception, and pregnancies subsequent to assisted reproductive techniques (ART) are common in obstetrical departments.

Although most pregnancies after IVF result in normal healthy outcomes, an increased risk for a number of obstetric and neonatal complications (such as preeclampsia, preterm delivery, lower average birth weight, and congenital malformations), compared to naturally conceived pregnancies, has been reported among singletons and twins [1-9]. This fact can be explained partly by the high rate of multiple pregnancies as a result of these procedures, and by advanced maternal age [10], but past research has also focused on the potential negative impact of micromanipulation techniques, extended culture systems, and medications used in the context of IVF/ICSI [11,12].

While there are many studies that compare pregnancies after ART with spontaneously conceived pregnancies, fewer data are available that evaluate the differences between IVF and ICSI-conceived pregnancies. A recent study from Norway found that IVF pregnancies were associated with an increased risk of iatrogenic preterm delivery compared to ICSI pregnancies [13].

The aim of the present study was to report systematically on the course of 334 IVF or ICSI-conceived pregnancies and to focus on the differences between both groups with regard to maternal and fetal outcome parameters.

## Methods

Between September 2003 and January 2009, 482 pregnant women with 722 fetuses, who had conceived via ART were referred to the Department of Obstetrics and Gynecology, Division of Obstetrics and Fetomaternal Medicine, Medical University of Vienna, Austria. Seventeen of the 722 fetuses were stillborn in the early gestational weeks, and, in 175 cases, there was insufficient information about the further course of pregnancy, and the status of birth and neonatal outcome, because patients were lost to follow-up. Therefore, after excluding cases of stillbirth and subjects lost to follow-up, a total of 334 pregnancies and 530 children could be included in our retrospective cohort study.

Outcome variables included maternal parameters: i.e., age; BMI; gravity; number of fetuses; pregnancy complications such as pregnancy-induced hypertension, preeclampsia, premature rupture of membranes, cervical insufficiency, and premature uterine contractions; and number of hospitalizations; and fetal parameters: i.e., gestational age; birth weight; birth height; pH of the umbilical artery; APGAR after one minute; APGAR after 10 minutes; congenital malformation; admission of the newborn to a neonatal intensive care unit (NICU); and death of the infant.

Institutional Review Board approval was obtained for the study, and women had given informed consent for the analysis of their data in the context of any retrospective evaluation. The present study was performed after approval of the responsible ethics committee. Ethikkommission der Universität Wien / Ethics commission of the Medical University of Vienna.

Statistical analysis was performed by the Center for Medical Statistics, Informatics and Intelligent Systems, Section for Medical Statistics of the Medical University of Vienna.

Descriptive results are given as mean ± standard deviation (or median and range) or frequencies and percentages. Spearman correlation coefficients were calculated.

Comparisons between IVF and ICSI pregnancies for variables that were considered maternal parameters were performed using t-tests (Wilcoxon-tests) or chi^2^-tests (Fisher’s exact tests). Comparisons between the two groups for fetal variables were performed using linear or generalized linear mixed models (ANOVA/ANCOVA).

In addition, based on correlations and information from the literature, some of the models were adjusted for other known influence factors and, hence, are analyzed using linear or generalized linear regression models.

As a secondary analysis, considering only singleton pregnancies, differences in gestational age between the two groups were calculated using the t-test (or Wilcoxon- test).

Analyses were performed using SAS 9.2 and SPSS 20 software. P-values <0.05 were considered statistically significant.

## Results

A total of 530 children of 334 mothers were included in our data analysis, 80 conceived via ICSI, and 450 via conventional IVF treatment.

In 155 cases (21 ICSI/ 134 IVF), we found a singleton pregnancy, in 160 cases (25 ICSI/135 IVF) twins, in 17 cases triplets (3 ICSI/ 14 IVF), and in one case a quadruplet after conventional IVF treatment.

The distribution between singleton and multiple pregnancies was not significantly different between IVF and ICSI.

Maternal characteristics and outcome parameters are given in Tables 1 and 2.

**Table 1:** Maternal characteristics and outcome parameters

**Variable**	**ICSI**				**IVF**				**p-value**
	**Mean**	**StdDev**	**Min**	**Max**	**Mean**	**StdDev**	**Min**	**Max**	
*Age*	33.31	5.20	23.0	48.0	33.71	4.92	20.0	49.0	0.60
*BMI*	25.24	4.63	18.2	37.5	24.55	4.52	17.6	46.8	0.35
	**Median**		**Min**	**Max**	**Median**		**Min**	**Max**	
*Gravity*	1		1	5	1		1	13	0.89
*Number of Fetuses*	2		1	3	2		1	4	0.10

Models are adjusted for correlated influence factors.

**Table 2:** Maternal characteristics and outcome parameters (continued)

**Variable**	**ICSI**		**IVF**		**p-value**
	**n**	**%**	**n**	**%**	
*Hypertension, Preeclampsia*	2	4.1	13	4.6	0.99
*Premature rupture of membranes, Cervical insufficiency, Premature contractions*	2	4.1	64	22.5	**0.0016**
Number of hospitalization events*					**0.0095**
*0*	43	87.8	199	70.1	
*1*	6	12.2	73	25.7	
*2*	0	0.0	11	3.9	
*4*	0	0.0	1	0.4	

Models are adjusted for correlated influence factors.

*unadjusted - should be adjusted for premature rupture of membranes,

Cervical insufficiency, and premature contractions, but the model did not converge then.

Women with IVF showed statistically significantly more frequent pregnancy complications, such as premature rupture of membranes, premature uterine contractions, or cervix insufficiency (p = 0.0016), and had to be hospitalized more often during the second and third trimester of pregnancy (p = 0.0095).

Neonatal outcome parameters are shown in Tables 3 and 4 (for all fetuses) and Tables 5 and 6 (results for singleton and multiple pregnancies given separately).

**Table 3:** Neonatal outcome parameters (all newborns, n = 530)

**Variable**	**ICSI**				**IVF**				**p-value**
	**Mean**	**StdDev**	**Min**	**Max**	**Mean**	**StdDev**	**Min**	**Max**	
*Gestational age*	34.04	4.56	23.00	42.00	35.70	4.00	21.00	42.00	**0.001**
*Birth height*	45.58	5.09	32.00	55.00	46.37	5.21	25.00	57.00	0.781
*Birth weight*	1973.7	812.67	408.00	3970.00	2345.3	799.00	323.00	4880.00	**0.008**
*NApH*	7.27	0.07	7.11	7.43	7.26	0.08	6.90	7.46	0.094
	**Median**	**Mean**	**Min**	**Max**	**Median**	**Mean**	**Min**	**Max**	**p-value**
*APGAR1*	8	7.49	0	9	9	8.13	0	10	**0.016**
*APGAR10*	10	8.68	0	10	10	9.31	0	10	**0.047**

Calculation concerning birth height is adjusted for the correlated influence factor, gestational age.

Calculation concerning birth weight is adjusted for the correlated influence factors, gestational age and birth height. Models are adjusted for correlated influence factors.

**Table 4:** Neonatal outcome parameters (all newborns, n = 530) (continued)

**Variable**	**ICSI**		**IVF**		**p-value**
	**n**	**%**	**n**	**%**	
*Fetal malformation*	3	4.7	4	0.9	0.081
*Admission to neonatal intensive care unit (NICU)*	30	47.6	123	29.6	**0.004**
*Infant death*	6	7.8	20	4.5	0.25

Models are adjusted for correlated influence factors.

**Table 5:** Neonatal outcome parameters (singletons, n = 155)

**Variable**	**ICSI**				**IVF**				**p-value**
	**Mean**	**StdDev**	**Min**	**Max**	**Mean**	**StdDev**	**Min**	**Max**	
*Gestational age*	36.48	4.72	25.00	42.00	38.23	3.06	27.00	42.00	0.114
*Birth height*	47.35	6.42	32.00	55.00	49.85	3.49	35.00	57.00	0.367
*Birth weight*	2583.8	925.7	710.00	3970.00	2990.8	728.4	875.00	4880.00	0.278
*NApH*	7.28	0.06	7.15	7.37	7.26	0.08	6.97	7.46	0.344
	**Median**	**Mean**	**Min**	**Max**	**Median**	**Mean**	**Min**	**Max**	**p-value**
*APGAR1*	9	7.90	1	9	9	8.55	3	10	**0.041**
*APGAR10*	10	9.35	1	10	10	9.81	4	10	**0.048**

Calculations concerning birth height are adjusted for the correlated influence factor, gestational age.

Calculations concerning birth weight are adjusted for the correlated influence factors, gestational age and birth height.

**Table 6:** Neonatal outcome parameters (multiples, n = 375)

**Variable**	**ICSI**				**IVF**				**p-value**
	**Mean**	**StdDev**	**Min**	**Max**	**Mean**	**StdDev**	**Min**	**Max**	
*Gestational age*	33.11	4.18	23.00	38.00	34.62	3.87	21.00	40.00	**0.009**
*Birth height*	44.62	3.98	37.00	52.00	44.74	5.08	25.00	52.00	0.915
*Birth weight*	1766.9	660.0	408.00	3170.00	2069.2	656.5	323.00	3376.00	0.149
*NApH*	7.27	0.07	7.11	7.43	7.26	0.08	6.90	7.40	0.098
	**Median**	**Mean**	**Min**	**Max**	**Median**	**Mean**	**Min**	**Max**	
*APGAR1*	8	7.35	0	9	9	7.95	0	10	**0.043**
*APGAR10*	10	8.44	0	10	10	9.10	0	10	**0.050**

Calculations concerning birth height are adjusted for the correlated influence factor, gestational age.

Calculations concerning birth weight are adjusted for the correlated influence factors, gestational age and birth height.

Mean gestational age was significantly lower in the ICSI group when including all pregnancies (p = 0.001, ƞ^2^p = .019). Moreover, the gestational age was statistically significantly lower in the group of multiple gestations (p < 0.001, ƞ^2^p = .080) compared to singletons, but also when comparing multiples after ICSI with multiples after IVF (p = 0.009, ƞ^2^p = .019). Focusing only on singleton pregnancies, we found no significant difference in gestational age.

We also determined gestational age categories to assess the risk of preterm delivery among deliveries and defined extremely preterm as < 28 weeks (age group 1), and moderately preterm from 28 to 34 weeks (age group 2), and compared the early age groups against children born after week 34 (age group 3). There was a statistically significant difference in distribution between ICSI with 7.9%, 46.05%, and 46.05% children, and IVF with 4.7%, 24.2%, and 85.5% children in the three age groups (p = 0.0039). Considering only singleton pregnancies, there were no children in the lowest age group, and 14.3% and 85.7% children in the higher groups after ICSI. In the IVF group, 0.8%, 8.1%, and 91.1% singletons were found in the three age groups. These distributions were not statistically significant (p = 0.62).

Birth weight was statistically significantly lower within the ICSI group (p = 0.008, ƞ^2^p = .016) when considering all fetuses; multiples had a lower birth weight compared to singletons in both groups, but there was no difference between IVF and ICSI-conceived pregnancies.

Mean APGAR 1 and APGAR 10 indices were slightly lower within the ICSI group for singletons as well as for multiples.

Admission to NICU was significantly more often observed within the ICSI group.

(χ^2^ = 8.13, p = 0.004); moreover, multiples had to be more often hospitalized compared to singletons (χ^2^ = 32.21, p < 0.001).

No differences between the groups could be found regarding the number of major congenital malformations and infant deaths.

Concerning the distribution of female and male sex between the neonates, we found no significant difference between the IVF and the ICSI group (IVF: 45.6% baby girls, ICSI: 50.6% baby girls).

High correlations, with correlation coefficients greater than 0.7, were found between the number of hospitalizations and premature rupture of membranes, gestational age and birth height, gestational age and birth weight, birth height and birth weight, APGAR1 and APGAR10, APGAR1 and infant death, and APGAR10 and infant death. Correlations between 0.4 and 0.7 could be found between gestational age and APGAR1, gestational age and APGAR10, gestational age and admission to the NICU, gestational age and infant death, birth height and APGAR1, birth height and APGAR10, birth height and admission to the NICU, birth height and infant death, birth weight and APGAR1, birth weight and APGAR10, birth weight and admission to the NICU, birth weight and infant death, number of fetuses and gestational age, and premature rupture of membranes and maternal hospitalization.

## Discussion

The number of pregnancies after ART is still increasing and there is some data showing that IVF/ICSI is associated with a higher rate of complications with regard to both the course of pregnancy and neonatal outcome. Of note, the potential negative impact of micromanipulation techniques should be considered, but only a few research studies have made precise distinctions between IVF and ICSI pregnancies [11,12,14].

Therefore, we systematically analyzed and compared 334 pregnancies and 530 children after IVF and ICSI, and discovered that the course of pregnancy is more complicated in IVF pregnancies (leading to more frequent maternal hospitalizations), whereas the primary fetal outcome (i.e., APGAR value after one minute and the necessity for admission to a NICU) seems to be better in this group than after ICSI treatment.

The significant association between IVF pregnancies and premature rupture of membranes, premature uterine contractions, and cervical insufficiency could be seen as concordant with the results of a smaller study that reported an increased preterm delivery risk in IVF versus ICSI-conceived pregnancies [15]. Mean gestational age at the time of delivery, however, did not differ between the two groups in our study, which supports data from Bonduelle et al., who found no disproportion in preterm delivery between pregnancies after the two major methods of ART. Preeclampsia, recently linked with decreased ovarian reserve [1], was not found to be overrepresented in our IVF group, although one might have suspected a relation between female subfertility (not male subfertility, leading to ICSI) and decreased ovarian reserve, and therefore, preeclampsia.

There were significant differences between the groups concerning gestational age, fetal birth weight, and mean APGAR levels, with lower levels for all variables in the ICSI group. Moreover, children in this group had to be admitted to the NICU more often.

A plausible explanation for this phenomenon is lacking; one might, however, speculate that early manipulation of the egg cell for ICSI could have a long-term negative impact.

When analyzing age groups, we found the risk of preterm delivery to be higher after ICSI treatment, but the fact that this observation no longer existed when focusing only on singleton pregnancies led to the conclusion that this might be predominantly due to multiple gestations within the ICSI group. Of note, the average gestational age in multiples was significantly lower in the ICSI group compared to the IVF group; this was not true of singleton pregnancies, as shown in Tables 5 and 6.

Concerning major fetal malformations, there was no statistically significant difference between the two groups, which is in accordance with other studies [14,16,17]. Wen et al. recently performed a meta-analysis that included more than 124000 ART pregnancies [12], and found no difference in the risk for malformations between children conceived by IVF and/or ICSI, although ART-conceived pregnancies were generally at increased risk for birth defects, compared to naturally conceived pregnancies (OR: 1.37). This is in line with the results of Pinborg et al., who suspected subfertility *per se* as a major risk factor for adverse perinatal outcomes after ART [18]. The fact, however, that, even in the same mother, an ART singleton has a poorer outcome than a non-ART sibling, leads to the presumption that factors related to the hormonal stimulation, embryo culture, or cryopreservation may also have a potentially negative impact on the offspring. In contrast, a smaller case-controlled study revealed no increase in congenital malformations in ICSI-conceived pregnancies compared to naturally conceived pregnancies [11]—possibly because of the small sample size.

A Chinese study analyzing birth defects in over 15400 children after ART found that the distribution of birth defects reflects the distribution in the general population, while the total frequency of birth defects was not significantly higher within the ICSI group, compared to insemination or conventional IVF [19].

In our ICSI group, there was one case of a heart malformation and two cases of facial malformation, and, in the IVF group, two cases of heart malformation, one case of facial malformation, and one case of omphalocele combined with malformation of the limbs.

Notably, the number of children after ICSI was unusually low (15%) in our collective. A possible explanation could be that all women whose data were analyzed in the present retrospective study were referred to our tertiary care center by their gynecologist for a distinct reason (e.g., multiple pregnancy, advanced maternal age, desire for first-trimester screening, wish for a second opinion concerning the mode of delivery), and women who undergo an ICSI procedure, typically performed for male subfertility, might be younger and more healthy, and therefore, less often referred to a tertiary care unit. However, age, BMI, and pre-existing diseases, such as hypertension, did not differ between the two groups in our study.

We have to admit, however, that the disproportionate underrepresentation of ICSI is an obvious limitation of our study.

Moreover, the “origin” of sperm for ICSI (ejaculated/frozen/after testicular sperm extraction/after epididymal sperm aspiration) was unfortunately not systemically noted in our data. A recent cohort study revealed, however, that the above-mentioned methods of operative sperm collection seem to be as safe as conventional ICSI, IVF, and natural conception with regard to neonatal outcome, including congenital malformations [20].

Another possible limitation of our study is the lack of an age-matched control group.

We decided, however, against creating a control group from our high-risk collective at the University clinic (only a few uncomplicated pregnancies are treated at our center) in order to avoid comparing one high-risk collective (e.g., women after preterm delivery or premature rupture of membranes in a past pregnancy), with another (St. p. ART) and preferred to use data concerning uncomplicated pregnancy outcomes from low-risk collectives reported in the literature.

## Conclusions

We systematically investigated a pre-defined group of Austrian women after conception via IVF/ICSI. We found that the course of pregnancies was more complicated after conventional IVF, whereas the primary fetal outcome seems to be better in this group, compared to ICSI treatment. Of course, our results must be interpreted with caution, as the study had a very skewed population, with underrepresentation of ICSI cases, and because the data were drawn exclusively from a tertiary care center. Further research that includes a larger cohort of ART pregnancies is required to confirm our hypothesis.

## Abbreviations

ART: Assisted reproductive techniques; i.e.: Id est; ICSI: Intra cytoplasmatic sperm injection; IVF: In vitro fertilization.

## Competing interest

The authors declare that they have no competing interest.

## Authors’ contributions

KN designed, analyzed, and reported the study. JO and DP analyzed the data. LS acquired the data presented in Tables 1, 2, 3, 4, 5 and 6. SF performed the statistical analysis. KW analysed, reported, and revised the study. All authors contributed to the study protocol, and read and approved the final manuscript.
